# The histopathological synovitis score is influenced by biopsy location in patients with knee osteoarthritis

**DOI:** 10.1007/s00402-021-03889-x

**Published:** 2021-04-10

**Authors:** Haider Mussawy, Jozef Zustin, Andreas M. Luebke, André Strahl, Veit Krenn, Wolfgang Rüther, Tim Rolvien

**Affiliations:** 1 Division of Orthopaedics, Department of Trauma and Orthopaedic Surgery, University Medical Center Hamburg-Eppendorf, Martinistrasse 52, 20246 Hamburg, Germany; 2 Institute of Histopathology Regensburg, Regensburg, Germany; 3 Department of Pathology, University Medical Center Hamburg-Eppendorf, Hamburg, Germany; 4 Institute of Pathology, Trier, Germany

**Keywords:** Synovitis, Histopathology, Krenn score, Osteoarthritis, Rheumatic disease

## Abstract

**Introduction:**

Osteoarthritis (OA) and rheumatoid arthritis (RA) represent the most common forms of arthritis, which are mainly caused by mechanical and inflammatory components, respectively. Determination of synovial inflammation in synovial biopsies via the histopathological Krenn score may be crucial for correct diagnosis and treatment. Specifically, it remains unclear whether synovitis scores differ among multiple biopsy locations within a single joint.

**Materials and methods:**

Eighty synovial samples were taken from four standardized regions of the knee in 20 patients (ten primary OA, ten secondary OA) undergoing total knee arthroplasty (TKA) or total synovectomy. The Krenn synovitis score (grade 0–9) was determined in a blinded manner by two expert pathologists in all biopsies. Next to the inter-rater reliability, we evaluated the agreement of the determined scores among the four biopsy locations within each knee.

**Results:**

The inter-rater reliability between the two pathologists was very high (Cohen’s kappa = 0.712; *r* = 0.946; ICC = 0.972). The mean synovitis score was significantly higher in knees with secondary than in primary OA (*p* = 0.026). Importantly, we found clear differences between the scores of the four different biopsy locations within the individual knee joints, with an average deviation of 10.6%. These deviations were comparable in knees with primary and secondary OA (*p* = 0.64).

**Conclusions:**

While we confirmed the synovitis score as a reliable and reproducible parameter to assess the histopathological synovitis grade in the knee, the considerable variability within the joint indicates that multiple synovial biopsies from different regions should be obtained to enable reliable results of the synovitis score.

## Introduction

The synovium is the target tissue of many inflammatory rheumatic diseases such as rheumatoid arthritis (RA), which represents the most prevalent form of autoimmune arthritis [1, 2]. The synovial membrane regulates synovial fluid and also plays an important role in the development of osteoarthritis (OA) [3, 4]. Synovitis leads to aseptic inflammation of the joint associated with swelling, pain and cartilage alterations [5] and constitutes an independent risk factor for knee OA [6]. The gold-standard method to assess synovitis is the histopathological analysis of tissue biopsy samples. This analysis not only allows a differentiation between inflammatory and non-inflammatory arthropathies, but also enables a better understanding of the disease pathophysiology and prognosis, as well as determination of treatment efficacy and identification of novel therapeutic targets [7]. Synovial tissue analysis may also enable personalized therapy in patients with RA [8, 9].

The Krenn histopathological synovitis score is a standardized and reproducible evaluation method, which is used in the differential diagnosis of joint diseases (i.e., inflammatory vs. non-inflammatory arthritis) [10]. This score takes into account three components of synovitis (lining layer hyperplasia, activation of resident cells (stroma) and inflammatory infiltrate). Scores range from 0 to 9, with scores of 1–4 indicating low-grade synovitis and scores of 5–9 indicating high-grade synovitis. Low-grade synovitis is usually associated with primary OA, whereas high-grade synovitis is associated with RA, psoriatic arthritis and other forms of arthritis [10]. While computer-assisted validation of the Krenn synovitis score has previously been demonstrated [11], the score has recently been extended with immunohistochemical markers to further improve sensitivity and specificity [12]. Furthermore, the score has been found to successfully differentiate various arthropathies accurately from normal tissue [13]. However, the site-specificity of the Krenn synovitis score within the same joint has not yet been evaluated in further detail.

Specifically, the minimum number of biopsies or regions for an accurate histological diagnosis has not yet been determined. Therefore, the aim of this study was to evaluate whether the synovitis score is dependent on the region of the knee from which the biopsy is obtained. In this regard, we also sought to determine the minimum number of biopsies required and the locations with the lowest and highest deviations.

## Methods

### Patients

**Fig. 1:**
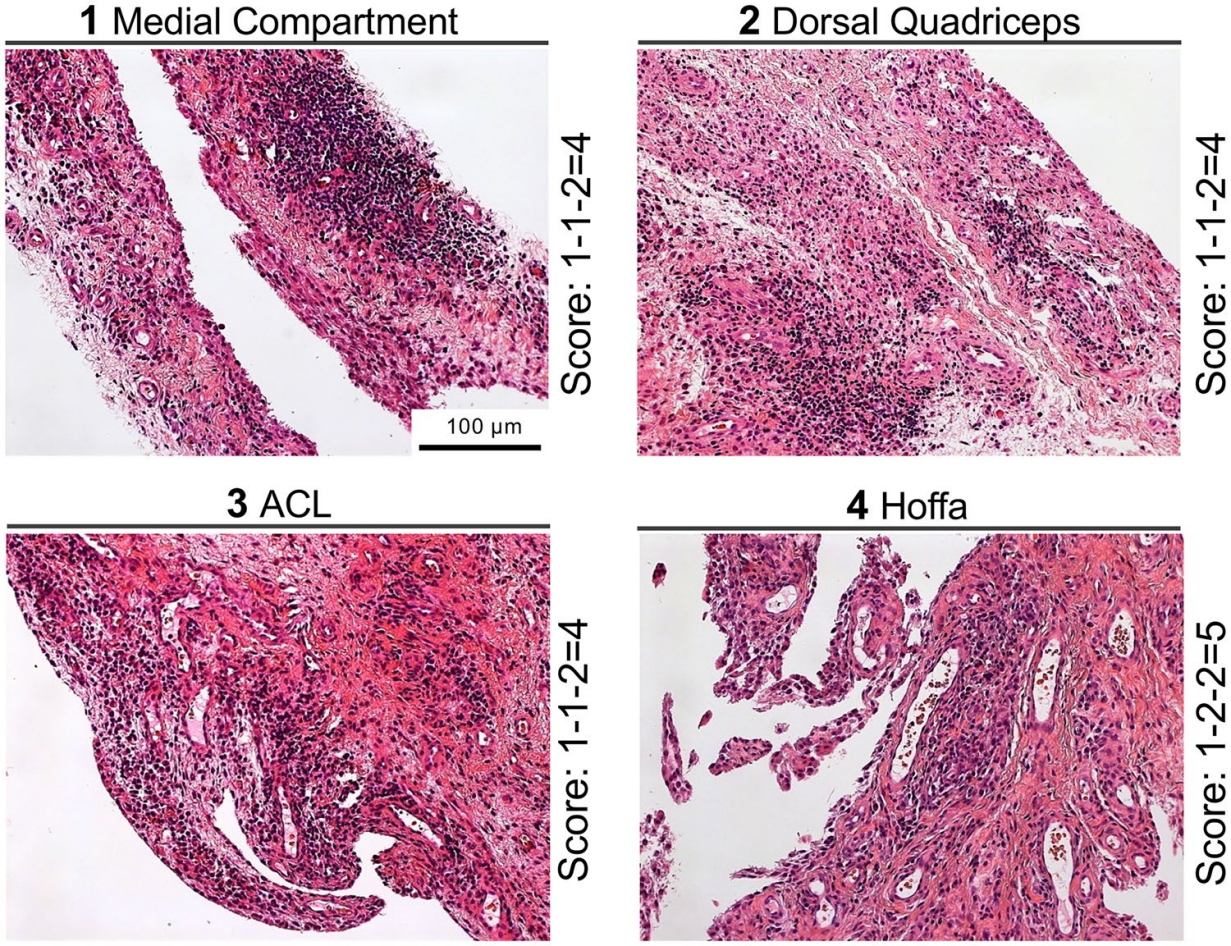
Exemplary images of tissue biopsies from the four locations within the knee joint with similar synovitis scores. 1: medial joint compartment, 2: the suprapatellar region (dorsal quadriceps), 3: the intercondylar region (around the anterior cruciate ligaments, ACL) and 4: the infrapatellar region (Hoffa fat pad), with their corresponding Krenn synovitis scores (H&E staining)

Eighty synovial samples were obtained intraoperatively from 20 patients as part of the standardized surgical procedure. Specifically, four biopsies were taken from ten consecutive patients with advanced stage primary OA and ten consecutive patients with advanced stage secondary OA undergoing total knee arthroplasty (*n* = 17) or synovectomy (*n* = 3) (Table 1). The synovial biopsies were collected from four standardized regions of each knee: the synovial region adjacent to the medial joint compartment [1], the suprapatellar region (dorsal quadriceps) [2], the intercondylar region (around the anterior cruciate ligaments, ACL) [3], and the infrapatellar region (Hoffa fat pad) [4] (Fig. 1). Of the ten patients with secondary OA, eight had been diagnosed with RA, while psoriatic arthritis (PsA) and ankylosing spondylitis (AS) were diagnosed in one of ten patients each. The mean age of the patients at the time of surgery was 66.8 ± 9.3 years. All biopsies were taken according to the recommendations of Najm et al. [8].

**Table 1:** Overview of the examined patients including diagnosis, type of surgery, synovitis score for each location and standard and maximum deviation between the four locations

No	Age	Diagnosis	Surgery	1 MC	2 SP	3 IC	4 Hoffa	Mean	SD	% SD	Max. D
1	68	p-OA	TKA	2	2	1	9	3.50	3.70	36.97	8
2	41	PsA	TAS	5	5	4	5	4.75	0.50	5.00	1
3	63	p-OA	TKA	3	3	2	2	2.50	0.58	5.77	1
4	69	p-OA	TKA	2	2	2	4	2.50	1.00	10.00	2
5	81	p-OA	TKA	2	3	3	0	2.00	1.41	14.14	3
6	73	p-OA	TKA	2	3	3	3	2.75	0.50	5.00	1
7	63	RA	TKA	2	3	2	4	2.75	0.96	9.57	2
8	64	RA	TKA	6	6	4	5	5.25	0.96	9.57	2
9	63	p-OA	TKA	2	3	2	3	2.50	0.58	5.77	1
10	71	p-OA	TKA	3	2	2	2	2.25	0.50	5.00	1
11	63	RA	TKA	2	1	2	2	1.75	0.50	5.00	1
12	54	RA	TKA	3	2	9	2	4.00	3.37	33.67	7
13	73	RA	TKA	4	4	3	3	3.50	0.58	5.77	1
14	54	RA	TAS	4	4	4	4	4.00	0.00	0.00	0
15	70	p-OA	TKA	2	2	3	1	2.00	0.82	8.16	2
16	77	p-OA	TKA	4	2	1	2	2.25	1.26	12.58	2
17	73	RA	TKA	5	4	6	5	5.00	0.82	8.16	2
18	77	RA	TKA	3	2	2	1	2.00	0.82	8.16	2
19	71	p-OA	TKA	3	4	3	1	2.75	1.26	12.58	3
20	68	AS	TAS	3	1	3	2	2.25	1.15	11.55	2
Mean	66.8			3.10	2.90	3.05	3.05	3.01	1.06	10.62	2.20
SD	9.3			1.21	1.29	1.82	2.07	1.07	0.91	9.11	1.96

*p-OA* primary osteoarthritis, *RA* rheumatoid osteoarthritis, *PsA* psoriatic arthritis, *AS* ankylosing spondylitis, *TKA* total knee arthroplasty, *TAS* trans-arthroscopic synovectomy, *MC* medial compartment, *SP* suprapatellar, *IC* intercondylar, *Hoffa* infrapatellar fat pad, *SD* standard deviation, *Max. D* maximum deviation

### Histology

**Fig. 2:**
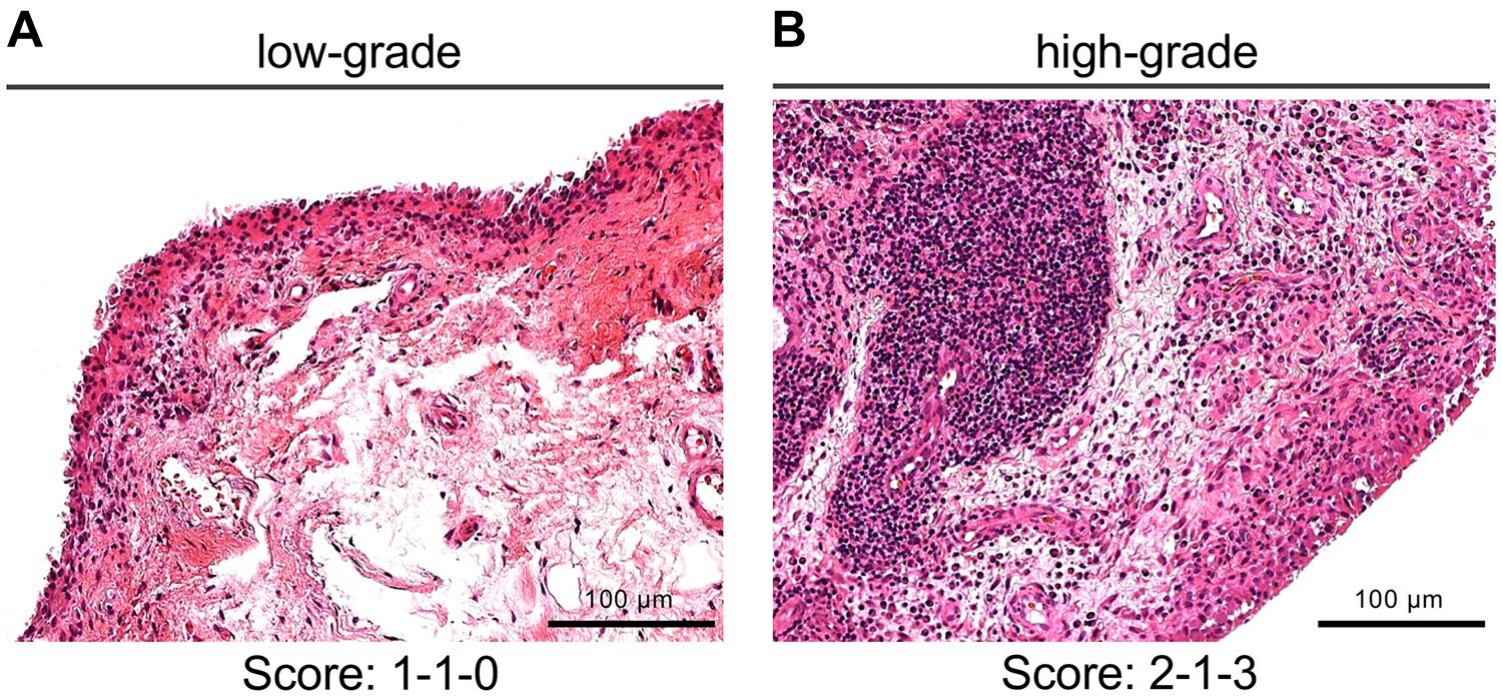
Differentiation of synovitis severity. Images of representative tissue biopsies showing **a** low-grade synovitis and **b** high-grade synovitis with corresponding Krenn synovitis scores consisting of lining layer hyperplasia, activation of resident cells (stroma) and inflammatory infiltrate (H&E staining)

Biopsy samples were fixed in 4% buffered formalin (pH 7.0) and embedded in paraffin for histological and immunohistochemical analyses. The Krenn synovitis score of each biopsy sample was determined in a blinded manner by two expert pathologists (JZ, AL) at two different institutions. There were no prior communications or meetings between the two pathologists regarding the use of scoring systems. Routine hematoxylin and eosin (H&E)-stained slides were graded based on three synovial membrane features (synovial lining cell layer, stroma cell density and inflammatory infiltrate) and the severity of the detected changes as described previously [14, 15]. Scores ranged from 0 to 9, with samples divided into those with low-grade (1–4) and high-grade (5–9) synovitis (Fig. 2).

### Statistical analysis

Statistical analyses were performed using SPSS 25 software (version 25.0, IBM, Armonk, New York, USA) and GraphPad Prism7 (GraphPad Software, La Jolla, CA). The inter-rater reliability, or the concordance of two or more evaluators, is an indicator of measurement accuracy and is essential to ensure that a measurement can be replicated independent of the particular evaluator [16]. Specifically, the Pearson correlation coefficient, the Cohen’s kappa coefficient and the intraclass correlation (ICC, two-way mixed effects, absolute agreement, multiple measurements) were calculated to determine agreement of synovitis scores between these raters. Based on 95% confident intervals (CIs), ICCs < 0.5, between 0.5 and 0.75, between 0.75 and 0.9 and > 0.90 indicated poor, moderate, good and excellent reliability, respectively [17]. ICCs were also calculated for the synovitis scores of biopsies among the four locations within each knee (medial, suprapatellar, intercondylar, Hoffa). Deviations were calculated to determine the locations that differed most and least. To compare the synovitis scores between primary OA and secondary OA, we used the unpaired two-sided *t* test. To compare the synovitis scores between the four biopsy locations, paired one-way analysis of variance (ANOVA) with Tukey’s post hoc analysis was performed. *P* values < 0.05 were considered statistically significant.

## Results

### Inter-rater reliability

**Fig. 3:**
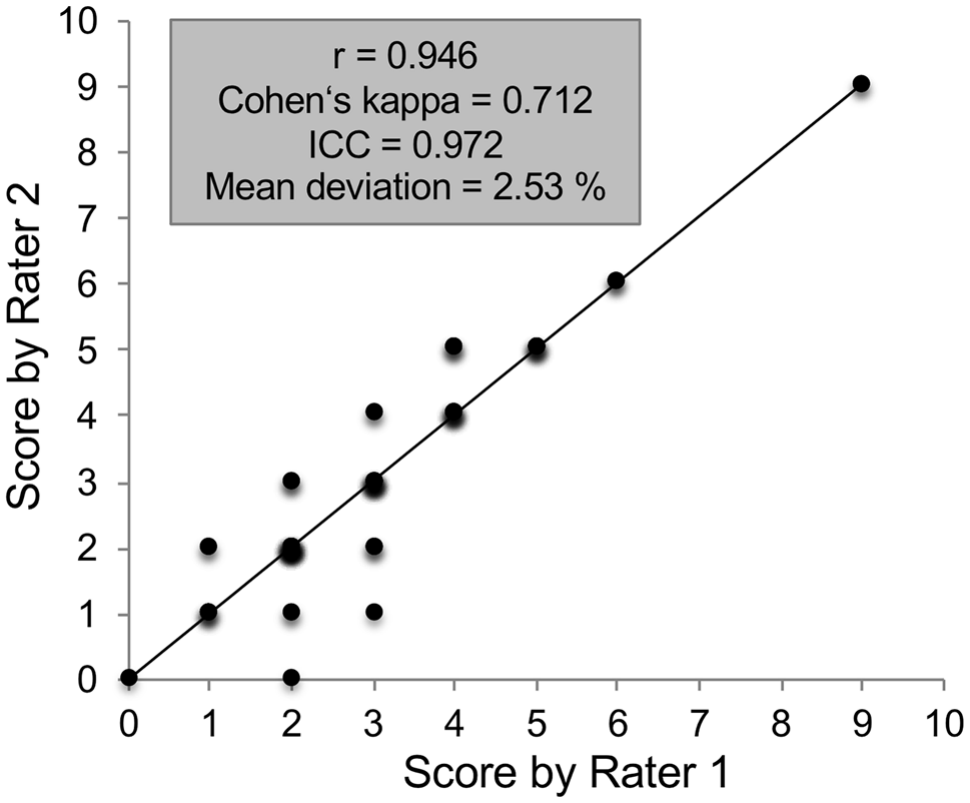
Concordance of the results of the synovitis scores assessed by two independent pathologists. The concordance was excellent, with an intraclass correlation (ICC) of 0.972 (95% CI 0.956–0.982, *p* < 0.001) and a high Pearson correlation coefficient (*r* = 0.946, *p* < 0.001), and a variance (*R*^2^) of 89.6%

A total of 80 synovial biopsy samples from 20 patients were analyzed in a blinded manner by two experienced, musculoskeletal pathologists (JZ, AL). Their concordance was excellent, with an ICC of 0.972 (95% CI 0.956–0.982, *p* < 0.001), Cohen’s kappa of 0.712, and a Pearson’s correlation coefficient of 0.946 (*p* < 0.001), corresponding to a variance of 89.6% (Fig. 3). Due to the high level of agreement, the data obtained from one rater (JZ) were used for further analysis.

### Site specificity of the synovitis score

Although no significant differences in the mean synovitis scores could be detected between any of the four biopsy locations (*p* > 0.05 for all comparisons, one-way ANOVA), the ratings of the 80 biopsy samples showed considerable variability among the four locations analyzed in 20 patients (Table 1). For example, in patient number 1, the Krenn synovitis score was 1 in the intercondylar region and 9 in the Hoffa fat pad, a result confirmed by both pathologists. Overall, we found a mean SD of 1.06 ± 0.91 (10.6%) among the four biopsy locations with a maximum of 2.2 ± 1.96 (Table 1). There were 7/20 (35%) patients, in which the SD was > 1 (≥ 10%). No consistent, specific clinical picture could be observed in these patients with the highest deviations. Furthermore, in 5/20 (25%) patients, part of the samples within the knee joint were scored in the range of high-grade synovitis (≥ 5), while others were scored in the area of low-grade synovitis (< 5).

The ICC calculated for tissue samples from all four regions was 0.579 (95% CI 0.151–0.817, *p* = 0.008). ICCs were lower when three (0.497; 95% CI 0.269–0.662, *p* < 0.001) or two (0.396, 95% CI 0.132–0.580, *p* = 0.003) samples were analyzed (Table 2). A further analysis to determine which localization differs most from the others revealed that the synovitis score of the Hoffa fat body differed most in both negative and positive directions from other tissue samples only in patients with primary OA (Mean deviation 0.6 ± 2.67, range 10). Patients with secondary OA showed a significantly smaller deviation (− 0.1 ± 0.88, range 3).

**Table 2:** Concordance of the histopathological synovitis score across all samples using intraclass correlation (ICC)

		95% CI		
	ICC	Lower	Upper	*p *value
All patients (*n* = 20)				
4 samples	0.579	0.151	0.817	0.008
3 samples	0.497	0.269	0.662	< 0.001
2 samples	0.396	0.132	0.580	0.003

*CI *confidence interval, *ICC* intraclass correlation

**Fig. 4:**
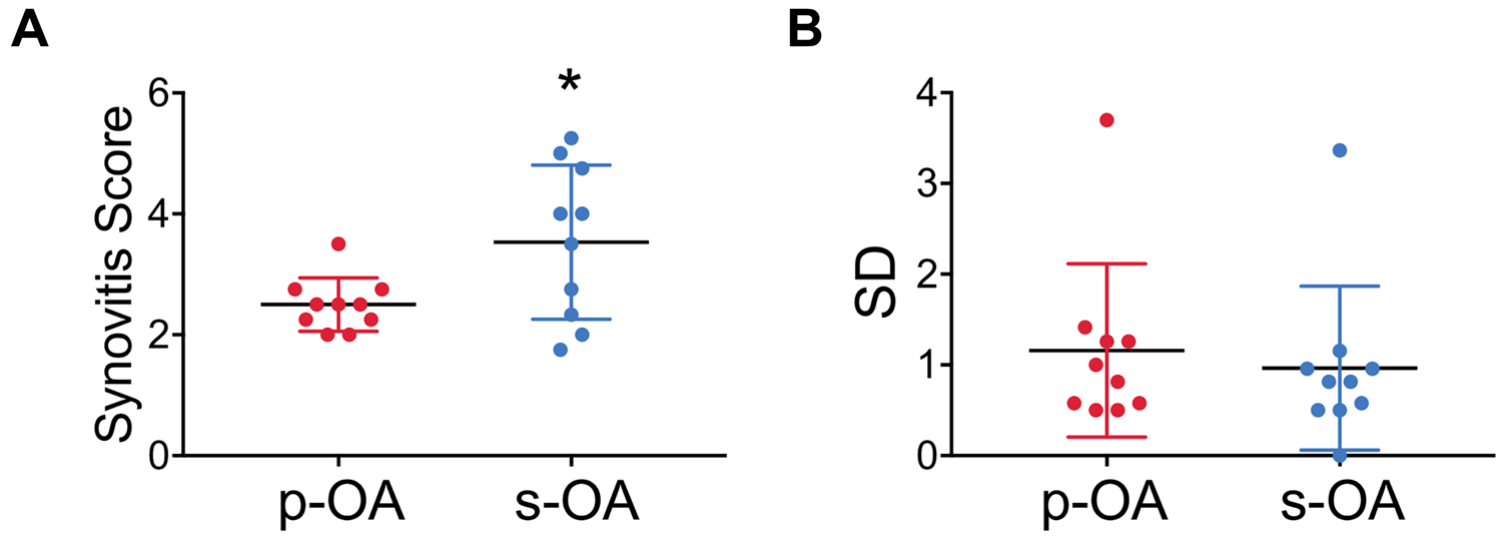
Mean synovitis score and standard deviation among the four biopsy locations in primary (p-OA) vs. secondary OA (s-OA). **a** Quantification of synovitis scores revealed higher mean values in s-OA compared to p-OA (*p* = 0.026). **b** There was no significant difference in the standard deviation (SD) of the synovitis scores of the four locations between p-OA and s-OA. *p < 0.05

Finally, although only 3/10 patients with secondary OA showed mean synovitis scores in the range of high-grade synovitis, the score was significantly higher in samples from patients with secondary than primary OA (*p* = 0.026; Fig. 4a). The standard deviation in the synovitis score among the four locations did not differ significantly in knees with primary and secondary OA (*p* = 0.64) (Fig. 4b).

## Discussion

In the present study, we demonstrated that the Krenn synovitis score varied considerably among different biopsy locations within the knee joint, indicating that the number of synovial biopsies obtained from each knee joint may be crucial. While it was previously shown that the Krenn synovitis score is highly reproducible [18], more detailed parameters of inter-rater reliability within the knee joint have not been reported. We here confirmed that the results of the Krenn synovitis score obtained by two expert pathologists have a very high inter-rater reliability, expressed by high correlation, ICC and Cohen’s kappa coefficients.

In addition to clinical, serological and imaging criteria, the synovitis score can be important in orthopaedic and rheumatic patients to make the correct diagnosis, especially in those with unclear (mono-)articular diseases [10]. As synovitis scores also provide information about inflammatory progression or regression during therapy, reliable scores have a high relevance in clinical practice [2]. In a previous study in patients with varus knee OA, the synovitis score was not associated with the histological OA grade; however, the authors concluded that the reason for the lack of significant associations may have been that they obtained synovial tissue biopsies only from the suprapatellar region [19]. In another study, MRI-based synovitis correlated with the histologic synovitis scores, but synovial tissue samples were obtained from a random location from the knee [20]. To address these limitations, standardization of tissue biopsies likely improves the comparability of assessments and reported results. In other words, given the inter-site variability of synovitis scores as detected in the present study, the individual assessment of four biopsies for each joint could enable more precise statements regarding the associations with, e.g., cartilage degeneration, MRI-based synovitis, and also treatment protocols.

Although most clinical guidelines do not include recommendations on the number of synovial biopsies for both clinical routine and translational research, the necessity of multiple biopsies from one joint to obtain a reliable synovitis score is a matter of ongoing debate. In this regard, a minimum of four synovial biopsies from different joint areas has been recommended in a previous consensus statement [8]. While another previous study reported moderate to strong correlations between the histological synovitis scores assessed in the medial, lateral and superior knee joint compartment, more detailed information on the variability of synovitis scores within these compartments was not provided [21]. Nonetheless, the authors emphasized the need for biopsies from these three knee compartments. For optimal interpretation of synovial tissue analysis results, a previous review article even recommended a minimum of six biopsies per joint [22]; however, this recommendation was only given in the context of research based on the observation to achieve a variance < 10% in the detection of T-cell activation [23], or to allow a determination of relevant differences in gene expression [24] in the synovial membrane. In the present study, evaluation of four biopsy samples from each joint yielded an ICC indicative of moderate reliability, with a decreasing reliability with fewer samples. Our results are compatible with the observation that the expression of cytokines varies within the same joint [25]. Hoffa fat pads showed the highest deviation in scores, a trend that was mainly observed in primary OA. Further studies including varying numbers of synovial biopsies are needed to confirm or contradict our results. Regarding the variability of synovitis within the knee joint, a previous MRI study in patients with OA demonstrated that synovial thickening typically appeared in the intercondylar region, in the Hoffa fat pad, or in the posterior joint margin [26]. Although we did observe considerable variations among the biopsy locations and the Hoffa fat pad did show the greatest deviations, there were no significant differences among biopsy locations. In particular, the synovitis score was not significantly higher in the intercondylar region or the Hoffa fat pad.

Indeed, tissue biopsy remains the gold standard option for determining the synovitis score. The usefulness of the synovitis score has also been demonstrated for other joints than the knee such as the shoulder (including traumatic diseases), where the score was able to distinguish between intra-articular and extra-articular pathologies [27]. Overall, synovial inflammation plays a critical role in the progression of primary and secondary OA and synovitis correlates with symptom severity [28]. Several anti-inflammatory agents that are successfully used for the treatment of OA have antisynovial effects expressed by reductions of synovitis scores [29]. An example for the local connection between joint damage and synovitis is the observation that medial abnormalities of perimeniscal synovium correlate with the severity of medial chondropathy in patients with medial OA [30]. While we confirmed that the synovitis score was significantly higher in patients with secondary OA than in patients with primary OA [31], our results underline that synovitis may occur in a site-specific manner in patients with both primary and secondary OA of the knee joint.

We acknowledge several limitations of the present study. The number of included patients was relatively small, suggesting the need for additional studies in larger numbers of patients and with long-term follow-up. Moreover, we analyzed patients at advanced stage OA, which is why no statements on the change in synovitis score along different OA grades can be made. Moreover, the RA patients suffered from secondary OA and possibly not (anymore) from high-grade synovitis at the time of biopsy, which was mirrored by the relatively low number of patients with high-grade synovitis. High-grade synovitis is defined by a synovitis score of ≥ 5, which was present in only 3/10 patients with secondary OA due to a rheumatic disease (4/10 when taking into account the highest value from each of the four biopsy locations). The primary reason for lower synovitis scores than expected may have been the advanced stage disease in these patients. In this regard, it is commonly accepted that most active rheumatic diseases are charactered by pronounced synovitis associated with bone erosions, which, due to repair mechanisms, can lead to secondary OA [32]. As recently discussed, only very few studies exist that compare inflammation with different severity stages in OA [20]. Compatible with our results, a previous study has compared local cytokine profiles in early stage and advanced stage knee OA and found lower levels of cytokines such as IL-15 in advanced stage OA [33]. The site specify of histopathological synovitis scores may also be different in patients at early stages of primary and secondary OA or other inflammatory joint diseases. Despite the known limitations of the present study, this work could reliably demonstrate the importance of the histopathological analysis of multiple tissue samples taken from one joint as well as the high reliability and clinical relevance of used scoring system if applied by an experienced pathologist.

In summary, the Krenn synovitis score is a reliable histopathological parameter to assess synovial inflammation in clinical practice. We demonstrated a relevant site specificity of the histopathologic changes within the knee joint with the Hoffa fat pad representing the least reliable location. Further studies are required to confirm the results of the present study in larger patient groups with more included joint disorders.

## Data Availability

The data that support the findings of this study are available from the corresponding author upon reasonable request.
